# Oestrogen induces epithelial‐mesenchymal transition in endometriosis via circ_0004712/miR‐148a‐3p sponge function

**DOI:** 10.1111/jcmm.15495

**Published:** 2020-07-15

**Authors:** Xin He, Nana Liu, Tianyi Mu, Dan Lu, Chanwei Jia, Shuyu Wang, Chenghong Yin, Lingyan Liu, Liying Zhou, Xiaowu Huang, Yanmin Ma

**Affiliations:** ^1^ Department of Obstetrics Beijing Obstetrics and Gynecology Hospital Capital Medical University Beijing China; ^2^ Center for Reproductive Medicine Department of Obstetrics and Gynecology Peking University Third Hospital Beijing China; ^3^ Department of Reproductive Medicine Beijing Obstetrics and Gynecology Hospital Capital Medical University Beijing China; ^4^ Department of Gynecology Beijing Obstetrics and Gynecology Hospital Capital Medical University Beijing China; ^5^ Department of Internal Medicine Beijing Obstetrics and Gynecology Hospital Capital Medical University Beijing China; ^6^ College of Pharmaceutical Sciences Capital Medical University Beijing China; ^7^ Department of Hysteroscopic Center Fuxing Hospital Capital Medical University Beijing China

**Keywords:** circ_0004712, EMT, endometriosis, miR‐148a‐3p, β‐catenin

## Abstract

Endometriosis is a common, chronic gynaecologic disease affecting up to 10% of women in their reproductive age and leading to pain and infertility. Oestrogen (E_2_)‐induced epithelial‐mesenchymal transition (EMT) process has been considered as a key factor of endometriosis development. Recently, the dysregulated circular RNAs (circRNAs) have been discovered in endometriosis tissues. However, the molecular mechanism of circRNAs on the E_2_‐induced EMT process in endometriosis is still unknown. Here, we demonstrated that circ_0004712 up‐regulated by E_2_ treatment in endometrial epithelial cells. Knock‐down the expression of circ_0004712 significantly suppressed E_2_‐induced cell migration activity. Meanwhile, we identified miR‐148a‐3p as a potential target miRNA of circ_0004712. Inhibited the expression of miR‐148a‐3p could recovered the effect of circ_0004712 knock‐down in E_2_‐treated endometrial epithelial. Furthermore, Western blot assay showed that E_2_ treatment could increase the expression and activity of β‐catenin, snail and N‐cadherin and reduce the expression of E‐cadherin. The expression and activity of β‐catenin pathway were recovered by circ_0004712 knock‐down or miR‐148a‐3p overexpression. Altogether, the results demonstrate that circ_0004712/miR‐148a‐3p plays an important role in E_2_‐induced EMT process in the development of endometriosis, and the molecular mechanism may be associated with the β‐catenin pathway. This work highlighted the importance of circRNAs in the development of endometriosis and provide a new biomarker for diagnosis and therapies.

## 1 INTRODUCTION

Endometriosis is a chronic disease in reproductive age women, which characterized as the ectopic growth of endometrial‐like tissues outside the uterine cavity, resulted in chronic pelvic pain and infertility.^1^ Due to the lack of effective biomarkers and treatment options, this chronic disease severely impairs patients’ quality of life and imposes a lot of economic burden.^2^ Therefore, exploring the effective markers for identifying the mechanisms related to exact pathogenesis of endometriosis is very important for improving the diagnosis and therapies.

Previous studies have demonstrated that abnormal oestrogen (E_2_) secretion is associated with the pathogenesis of endometriosis.^3^, ^4^, ^5^ Using aromatase inhibitor to suppress the activity of aromatase, which is a key enzyme to produce oestrogen, has been demonstrated to be an effective treatment for this disease.^6^ However, the molecular mechanism of E_2_ on the development of endometriosis has not been fully clarified. Recent study found that E_2_ could induce epithelial‐mesenchymal transition (EMT) process during the development of endometriosis.^7^

Epithelial‐mesenchymal transition is a biological process that promotes the polarized epithelial cell to process a mesenchymal phenotype, in which the epithelial cell obtain the ability of migration, invasion and re‐localization.^8^ The abnormal activation of EMT programs is considered as a key factor in tumour invasiveness and metastasis, and other pathological processes.^9^ Many studies revealed that an enhanced EMT‐like process was occurred in the establishment of ovarian endometriosis,^10^, ^11^, ^12^ but the molecular mechanism of oestrogen on inducing EMT process of endometrial epithelial cells still unknown.

Circular RNAs (circRNAs) are a number of non‐coding RNAs, which have been considered as a gene regulator at transcriptional or post‐transcriptional level.^13^ Because of its structure, it can stable expressed in cells and often act as miRNAs inhibitors by sponging miRNAs. Therefore, circRNAs could be potential biological regulators for recognizing the molecular mechanisms of disease and finding effective diagnostic biomarkers or therapeutic targets.

Previous study identified that circ_0004712 was significantly up‐regulated in endometriosis,^14^ but the biological functions of circ_0004712 still unknown. The aim of present study was explored the potential functions of circ_0004712 in the process of E_2_‐induced EMT in endometriosis development.

## 2 MATERIALS AND METHODS

### 2.1 Cell culture, treatment and transfection

Ishikawa cell and End1/E6E7 cell was obtained from American Type Culture Collection (ATCC; Manassas, VA, USA). Cells were cultured in Dulbecco's modified Eagle's medium (DMEM, Gibico, Carlsbad, CA, USA) supplemented with 10% foetal bovine serum (FBS, Gibico, USA), 50 U/mL penicillin and 50 mg/mL streptomycin. Cells maintained at 37°C in a 5% CO_2_ humidified incubator.

E_2_ was purchased from Sigma (E‐2758) and dissolved in dimethyl sulfoxide (DMSO). Cells were treated with different concentrations (0, 10^−12^, 10^−10^, 10^−8^ and 10^−6^ mol/L) of E_2_ and incubated 48 hours. The cells were incubated with serum‐free medium for 24 hours before E_2_ treatment.

The small interfering RNAs (siRNAs) targeting to the circ_0004712 (5′‐AACCTATATCAGGTACAACAT‐3′), miR‐148a‐3p mimics, miR‐148a‐3p inhibitor, miRNA negative control (NC) were designed and synthesized by Genepharma (Shanghai, China). Cells were transfected using Lipofectamine 2000 Reagent (Invitrogen, Carlsbad, CA, USA) according to the manufacturer's protocol.

### 2.2 qPCR

Total RNAs were isolated from treated cells by Trizol reagent (Invitrogen, Carlsbad, CA, USA). The first strand of cDNA was obtained by PrimeScript RT Master Mix (Takara, Dalian, China). Specific gene expression was detected by SYBR Premix Ex Taq (Takara) using ABI PRISM 7500 Sequence Detection System (Life Technologies, Grand Island, NY, USA). The relative expression data were normalized and analysed by the equation $2^{-\Delta \Delta C_{t}}$ . The primers are shown in Table 1. GAPDH was used for circ_0004712 normalizing, and U6 were used for miR‐148a‐3p normalizing.

**TABLE 1:** The primers used for qPCR

Symbol	Sequences (5′‐3′)
GAPDH‐F	ACAACTTTGGTATCGTGGAAGG
GAPDH‐R	GCCATCACGCCACAGTTTC
circ‐F	AGCAGCACACGATGTGGA
circ‐R	CCTTTTTCTTGGTGCCAATC
miR‐F	GCGCTCAGTGCACTACAGAA
miR‐R	AACTGGTGTCGTGGAGTCGGC
miR‐RT	GTCGTATCCAGTGCAGGGTCCGAGGTATTCGCACTGGATACGACACAAAG
U6‐F	CTCGCTTCGGCAGCACA
U6‐R	AACGCTTCACGAATTTGCGT

### 2.3 Transwell assay

Cell migration was tested by 24‐well transwell chambers containing polycarbonate filters with a pore size of 8 μm (Corning Costar, USA). Before culture, cells were resuspended in 200 μL serum‐free DMEM at the concentrate of 10^5^ Cells/mL. Then, cells were seeded on the upper parts of the 24‐well plate, which contains 500 μL DMEM with 20% FBS at lower parts, and incubated at 37°C. The cells were fixed and stained in a 0.1% crystal violet solution for 15 minutes after 24 hours incubation. The stained migrated cells on the underside were photographed using microscopy (Nikon, Japan).

### 2.4 Due‐luciferase assay

The sequences of wild‐type circ_0004712 (circ‐WT), mutation circ_0004712 (circ‐Mut), wild‐type of 3′‐UTR of SOS2 (SOS2‐WT), mutation of 3′‐UTR of SOS2 (SOS2‐Mut) and were cloned into psiCHECK‐2 vector (Promega, Madison, WI, USA), respectively. The plasmid was co‐transfected with miR‐148a‐3p mimics or NC into cells for 48 hours. The luciferase activities were detected using the Dual‐Glo Luciferase Assay System (Promega). Firefly luciferase was used as a reporter gene for normalized control.

### 2.5 Western blot assay

Treated cells were lysed in radio‐immunoprecipitation assay (RIPA) buffer (Beyotime, Hangzhou, China) supplemented with PMSF (Sigma) for total protein extracting. The concentration of total protein was quantified using BCA protein assay kit (Beyotime). Equal protein (60 μg) was separated by 10% SDS‐PAGE and transferred onto a PVDF membrane (0.45 μm; Millipore). Following blocking with 5% non‐fat milk, the membrane was incubated overnight at 4°C with E‐cadherin (1:1000; ab40772, Abcam), N‐cadherin (1:1000; ab18203, Abcam), β‐catenin (1:1000; ab16051, Abcam), p‐β‐catenin (1:1000; ab11350, Abcam), Snail (1:1000; ab167609, Abcam), SOS2 (1:1000; ab154999, Abcam) and GAPDH (1:4000; ab181602, Abcam), then secondary antibodies at room temperature for 1 hour. After that, the band was exposed by ECL system (Thermo Fisher Scientific, USA) and analysed by Quantity One software (Bio‐Rad, San Diego, CA, USA).

### 2.6 Statistical analysis

All statistical analyses were performed using GraphPad Prism 8.0 (GraphPad Software Inc). Unpaired two‐sided *t* test was performed for analysing the between‐group differences. The data were expressed as mean ± SD *P* values of less than 0.05 were considered statistically significant. All experiments for statistical analyses were repeated for triple times.

## 3 RESULTS

### 3.1 circ_0004712 up‐regulated by E_2_ treating in endometrial epithelial cells

**FIGURE 1:**
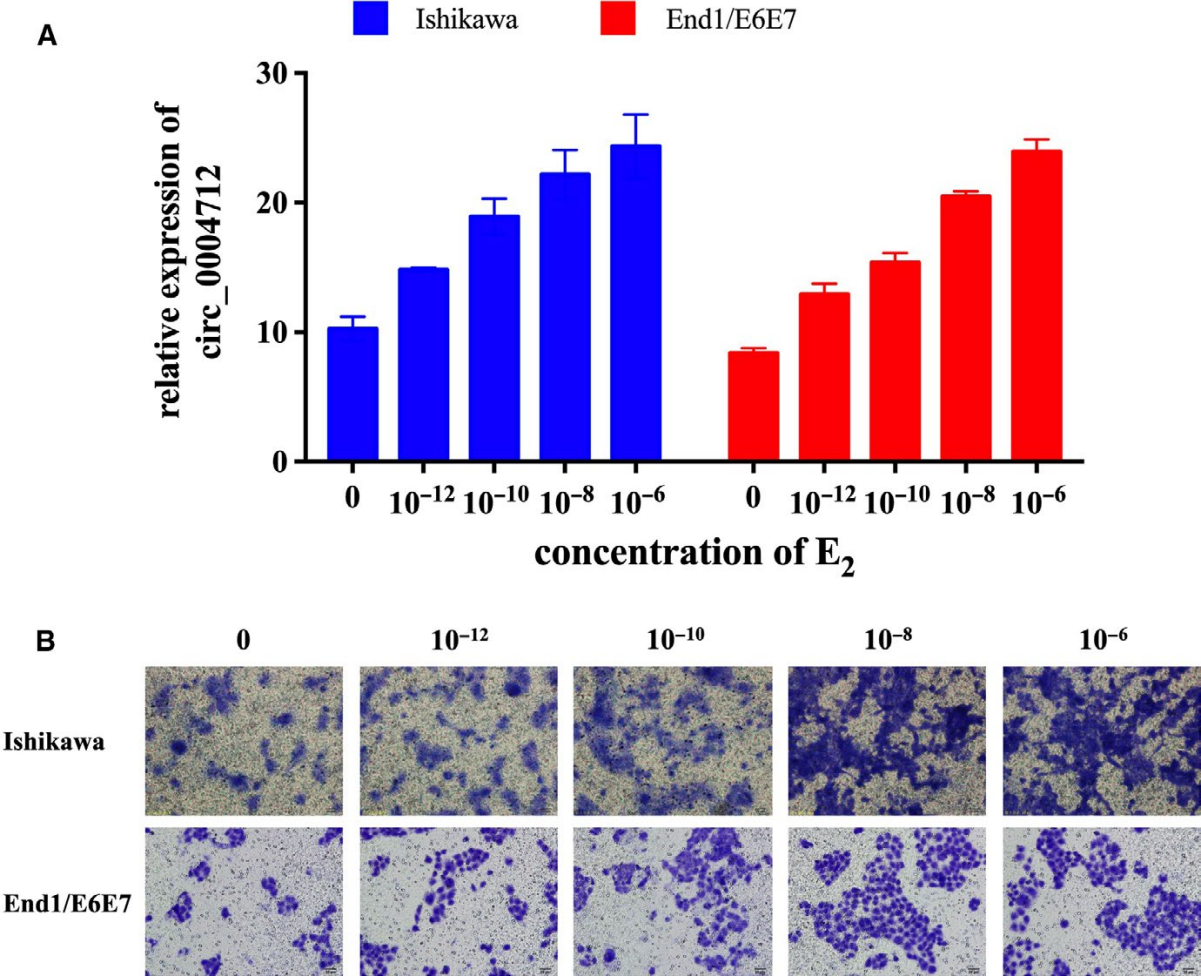
Effect of E_2_ on the expression of circ_0004712 and cell migration. A, The expression of circ_0004712 in E_2_‐treated Ishikawa and End1/E6E7 cells was tested by qPCR. Results show as mean ± SD. B, Cell migration of Ishikawa and End1/E6E7 cells after E_2_ treatment was analysed by transwell assay

To explore the relationship between E_2_‐induced EMT and circ_0004712 expression, we preformed different concentration of E_2_ (10^‐12^, 10^‐10^, 10^‐8^, 10^‐6^mol/L) treatment in endometrial epithelial cell lines. After 48 hours treatment, the expression of circ_0004712 was significantly up‐regulated as a dose‐dependent manner in both of Ishikawa and End1/E6E7 cells (Figure 1A). Transwell assay showed E_2_ treatment markedly induced the migration activity as a dose‐dependent manner (Figure 1B).

### 3.2 circ_0004712 promotes cell migration in endometrial epithelial cells after E_2_ treatment

**FIGURE 2:**
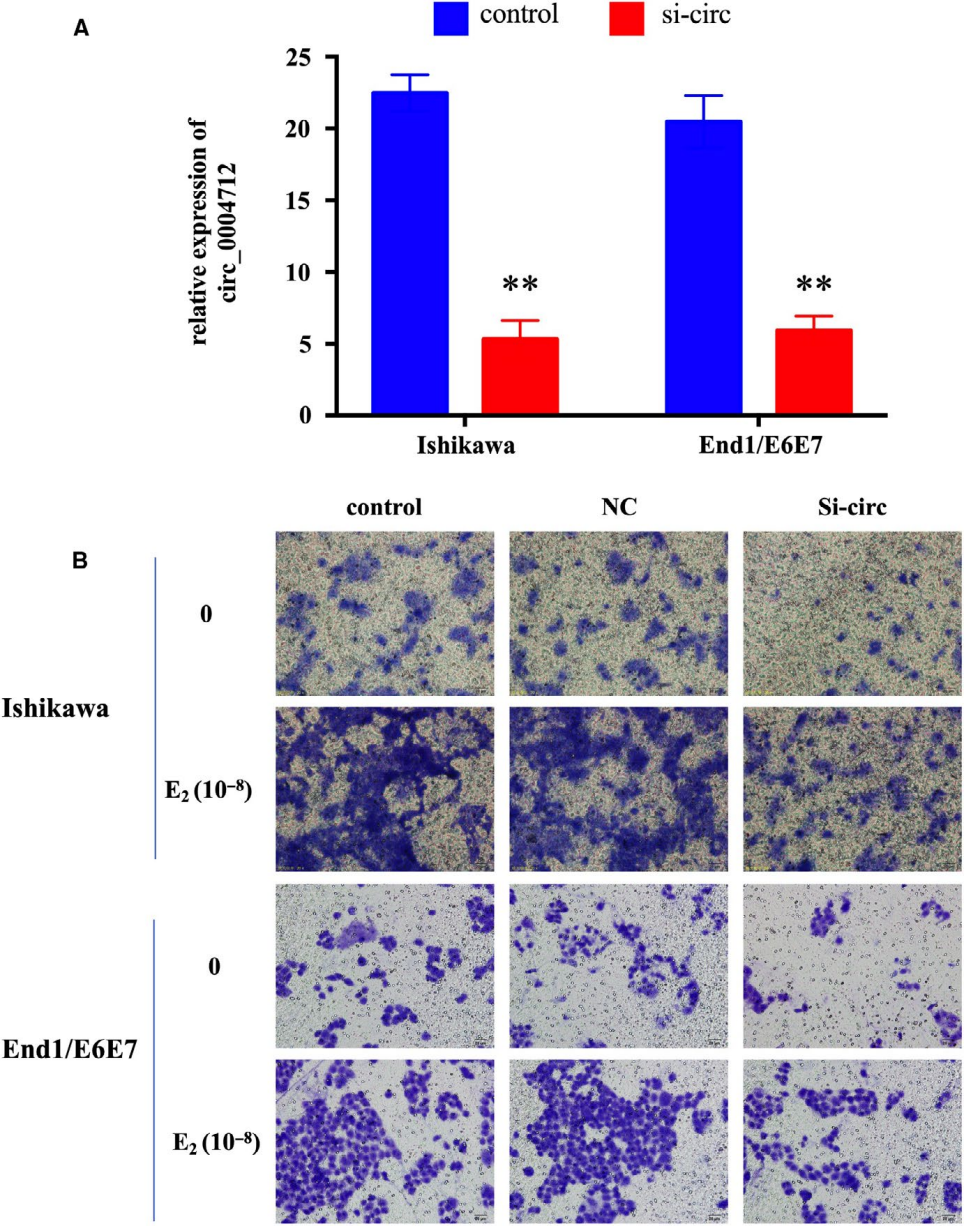
Effect of circ_0004712 on cell migration. A, The expression of circ_0004712 in Ishikawa and End1/E6E7 cells after for specific siRNA for circ‐0004712 (si‐circ) or negative control siRNA (NC) transfection was tested by qPCR. Results show as mean ± SD. **Means *P* < 0.05. B, Cell migration of E_2_ treated or untreated Ishikawa and End1/E6E7 cells after si‐circ or NC transfection was analysed by transwell assay

To study the potential biological effects of circ_0004712 on E_2‐_induced EMT process, we synthesized a specific interference RNA oligonucleotide (si‐circ) to knock down endogenous expression of circ_0004712 in endometrial cells after E_2_ (10^‐8^ mol/L) treatment. qPCR results showed the expression of circ_0004712 were significantly decreased by si‐circ transfection in E_2_‐treated Ishikawa and End1/E6E7 cells (Figure 2A). Transwell assay showed that knock‐down circ_0004712 significantly suppressed E_2_‐induced cell migration activity (Figure 2B). Overall, the results suggested that high expression of circ_00471 was related with E_2_‐induced EMT progress.

### 3.3 circ_0004712 targeted to miR‐148a‐3p

**FIGURE 3:**
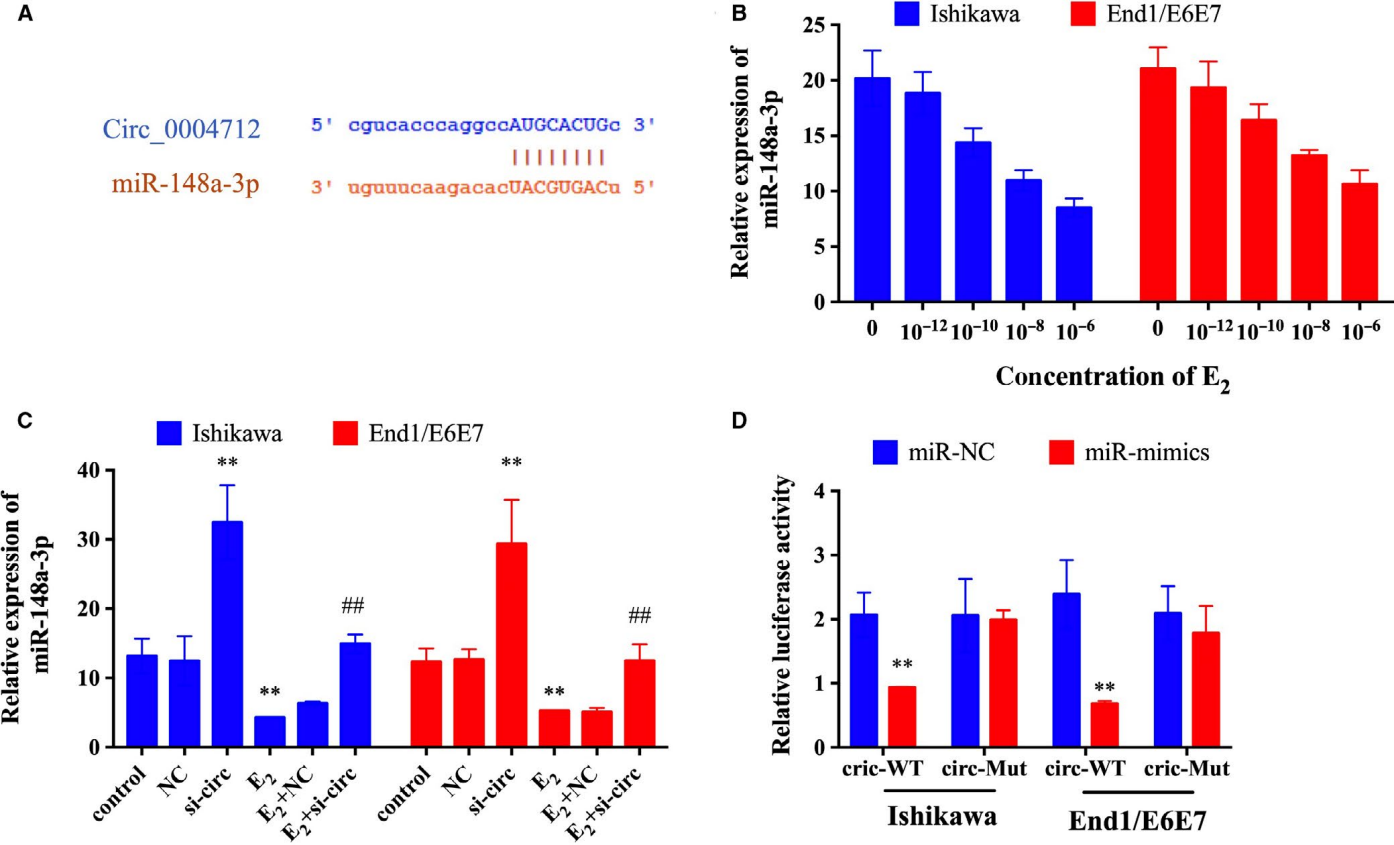
Circ_0004712 directly suppresses the expression of miR‐148a‐3p. A, The target sites of circ_0004712 and miR‐148a‐3p. B, The expression of miR‐148a‐3p in E_2_‐treated Ishikawa and End1/E6E7 cells was tested by qPCR. C, The expression of miR‐148a‐3p in E_2_‐treated orE_2_‐untreated Ishikawa and End1/E6E7 cells after for si‐circ or NC transfection was tested by qPCR. D, Relative luciferase activity in Ishikawa and End1/E6E7 cells transfected with miR‐NC or miR‐mimics and circ‐wild‐type (wt) or circ‐mutation (mut), respectively. Results show as mean ± SD. **Means compared with control group, *P* < 0.05. ##Means compared with E_2_‐treated group, *P* < 0.05

Many evidences revealed that circRNAs could sponge miRNAs to regulate the expression of the target genes. Thus, we predicted the potential target miRNAs of circ_0004712 by bioinformatics. MiR‐148a‐3p have a binding site to circ_0004712 (Figure 3A), and the expression of miR‐148a‐3p was significantly down‐regulated after E_2_ treatment as a dose‐dependent manner (Figure 3B). Furthermore, the expression of miR‐148a‐3p was significantly reduced by circ‐000471 knocking down (Figure 3C). The dual‐luciferase assay further demonstrated the directly binding site between circ_0004712 and miR‐148a‐3p (Figure 3D). Compared with control group, miR‐148a‐3p significantly reduced the relative luciferase activity, and no significant effect on luciferase activity when co‐transfected with mutated plasmids. These results suggested that circ_0004712 could bind to miR‐148a‐3p to modulate the E_2_‐induced EMT process in endometrial epithelial cells.

### 3.4 circ_0004712/miR‐148a‐3p regulated EMT process via β‐catenin pathway

**FIGURE 4:**
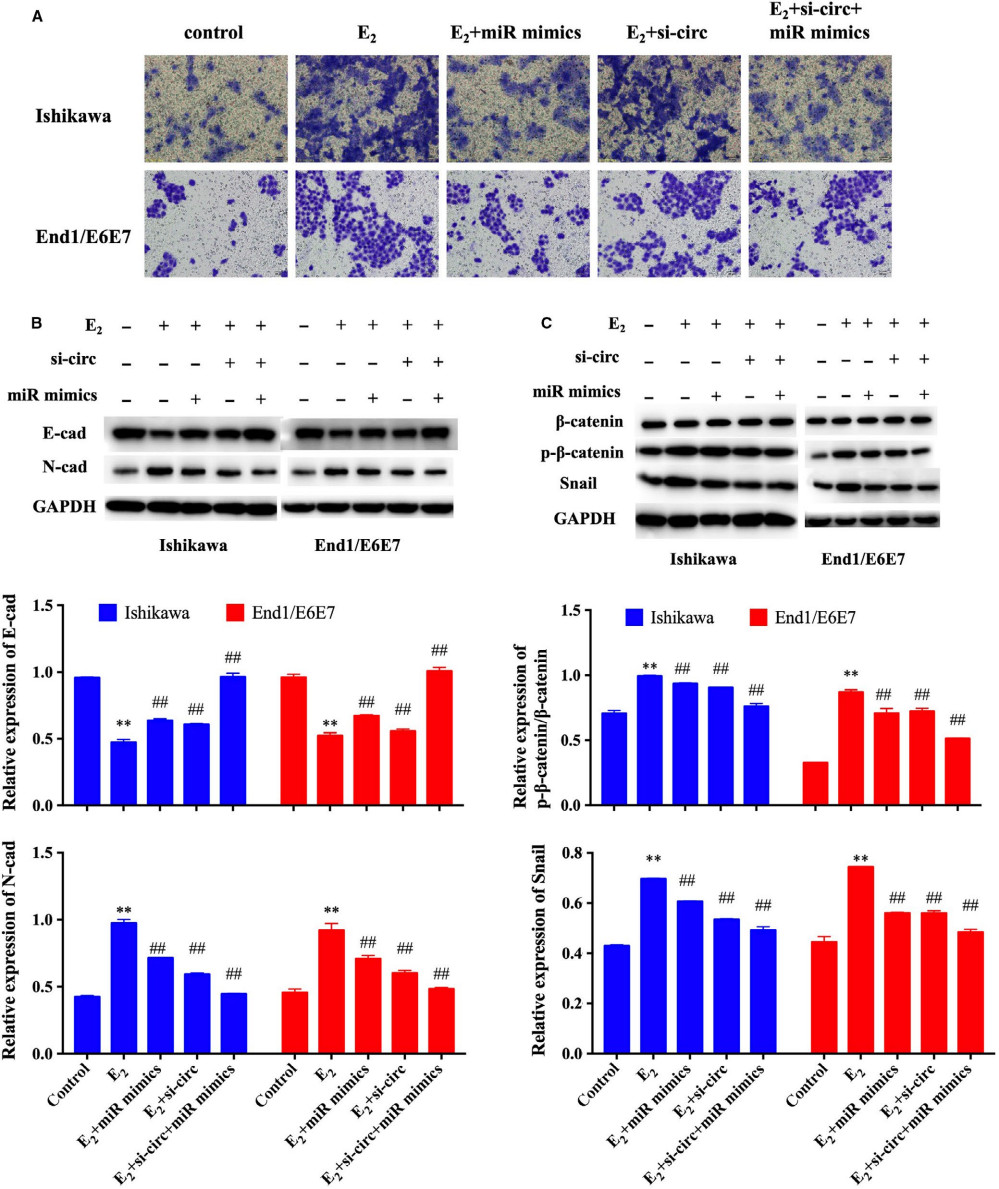
Effect of circ_0004712/miR‐148a‐3p on cell migration. A, Cell migration of E_2_‐treated orE_2_‐untreated ishikawa and End1/E6E7 cells after NC, si‐circ and/or miR mimics transfection was analysed by transwell assay, respectively. B, Protein expression of E‐cadherin and N‐cadherin in E_2_‐treated orE_2_‐untreated ishikawa and End1/E6E7 cells after NC, si‐circ and/or miR mimics transfection was analysed by Western blot assay. C, Protein expression of β‐catenin, p‐β‐catenin and Snail in E_2_‐treated or E_2_‐untreated ishikawa and End1/E6E7 cells after NC, si‐circ and/or miR mimics transfection was analysed by Western blot assay. Results show as mean ± SD. **Means compared with control group, *P* < 0.05. ##Means compared with E_2_‐treated group, *P* < 0.05

To further study the molecular mechanism of circ_0004712/miR‐148a‐3p on E_2_‐induced EMT process, cells were treated with E_2_ and transfected with si‐circ and/or miR‐148a‐3p mimics, respectively. Transwell assay revealed that inhibiting the expression of circ_0004712 or increasing the expression of miR‐148a‐3p could significantly suppress the migration capacity in E_2_‐treated endometrial epithelial cells (Figure 4A). Furthermore, the effect of circ_0004712 knock‐down on cell migration could be recovered by miR‐148a‐3p inhibitor. Western blot assay showed that E_2_ treatment could increase the expression and activity of β‐catenin pathway and N‐cadherin and reduce the expression of E‐cadherin (Figure 4B). The expression and activity of β‐catenin pathway were recovered by si‐circ or miRNA mimics transfecting (Figure 4C). These results suggested that circ_0004712 and miR‐148a‐3p had opposite effects on EMT process, and circ_0004712 could suppress the expression of miR‐148a‐3p. The molecular mechanism of circ_0004712/miR‐148a‐3p on the E_2_‐induced EMT process may be associated with the β‐catenin pathway.

### 3.5 miR‐148a‐3p targets to SOS2

**FIGURE 5:**
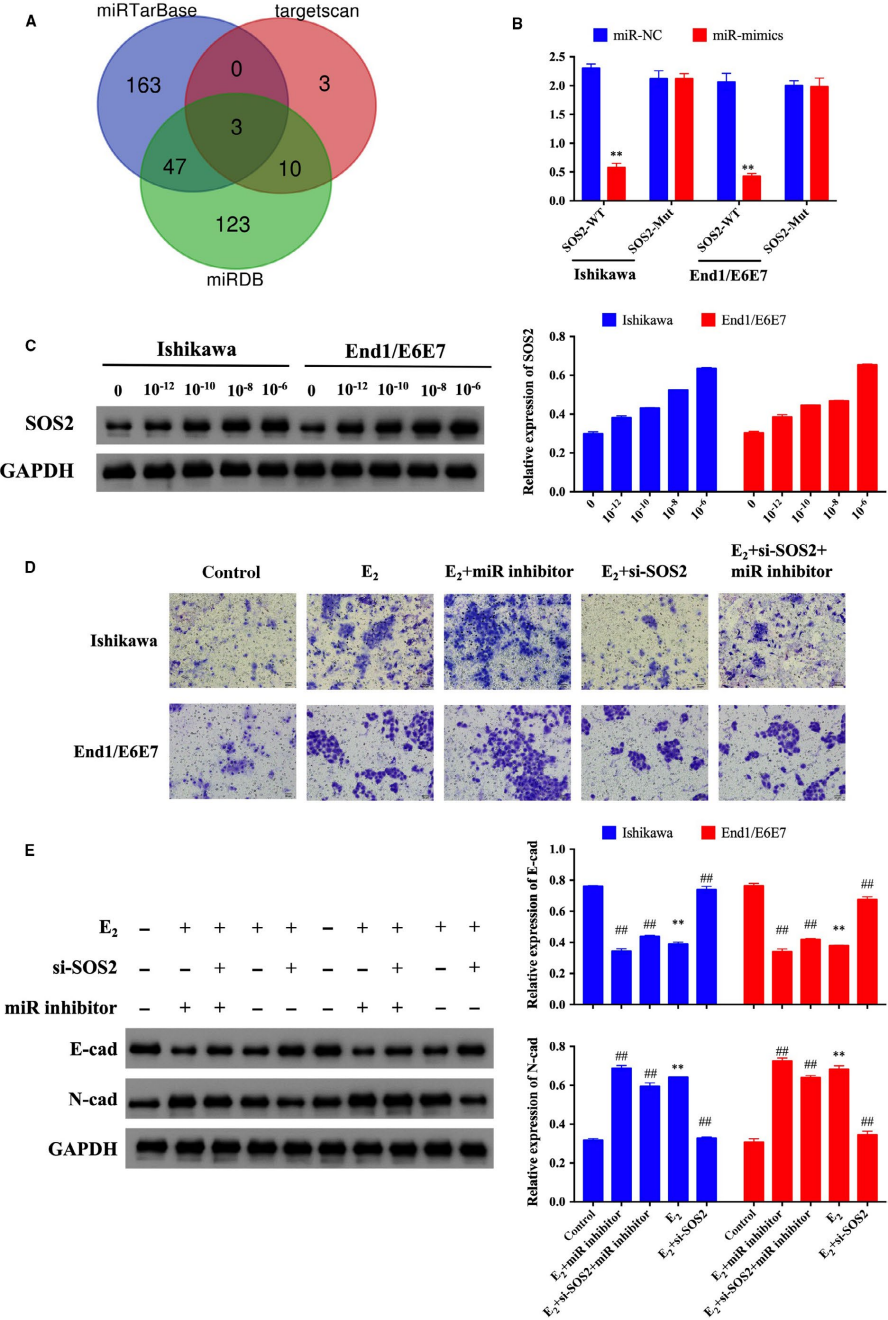
miR‐148a‐3p directly suppresses the expression of SOS2. A, Venn diagram showed overlapped target genes of miR‐148a‐3p in three databases. B, Relative luciferase activity in Ishikawa and End1/E6E7 cells transfected with miR‐NC or miR‐mimics and SOS2‐wild‐type (wt) or SOS2‐mutation (mut), respectively. C, The protein expression of SOS2 in E_2_‐treated Ishikawa and End1/E6E7 cells was tested by Western blot. D, Cell migration of E_2_‐treated or untreated Ishikawa and End1/E6E7 cells after NC, si‐SOS2 and/or miR inhibitor transfection was analysed by transwell assay, respectively. E, Protein expression of E‐cadherin and N‐cadherin in E_2_‐treated or E_2_‐untreated Ishikawa and End1/E6E7 cells after NC, si‐SOS2 and/or miR inhibitor transfection was analysed by Western blot. Results show as mean ± SD. **Means compared with control group, *P* < 0.05. ##Means compared with E_2_‐treated group, *P* < 0.05

miRNAs could bind to the 3′‐UTR of target genes. Thus, we predicted the target genes of miR‐148a‐3p through three databases (miRTarBase, targetscan7.2, and miRDB) and found three common genes (ARL8B, GLRX5 and SOS2) in these three databases (Figure 5A). In these three genes, SOS2 (Son of sevenless 2) was associated with EMT process.^15^ Thus, we tested the expression of SOS2 in E_2_‐treated Ishikawa and End1/E6E7 cells. The results of Western blot showed the expression of SOS2 was significantly increased after E_2_ treatment as a dose‐dependent manner (Figure 5C). The dual‐luciferase assay further demonstrated the directly binding site between miR‐148a‐3p and SOS2 (Figure 5B). Transwell assay revealed that knock‐down the expression of SOS2 could significantly suppress the migration capacity in E_2_‐treated endometrial epithelial cells and recovered the effect of miR‐148a‐3p inhibitor on the cell migration (Figure 5D). To further explore the effect of miR‐148a‐3p/SOS2 on the regulating the EMT process, we tested the expression of N‐cadherin and E‐cadherin. The results of Western blot showed that the expression of N‐cadherin and E‐cadherin were recovered by si‐SOS2 transfecting (Figure 5E). These results suggested that SOS2 was the target of miR‐148a‐3p involved in the circ_0004712‐mediated E_2_‐induced EMT process in endometrial cells.

## 4 DISCUSSION

Endometriosis is a common, chronic gynaecologic disease affecting women in their reproductive age and leading to pain and infertility.^16^ Identifying the accrue biomarkers and specific therapeutic targets for the early diagnosis and treatment is immediately needed. Increasing evidences have highlighted circRNAs as important molecular biomarkers and gene regulators. Recently, the expression profile of circRNAs in endometriosis has been reported,^14^, ^17^ and these studies indicated that dysregulated circRNAs might be potential molecular targets for clinical diagnosis and therapy. However, the molecular mechanism of these circRNAs is still unclear. Here, we studied the roles of an up‐regulated circRNAs (circ_0004712) on the E_2_‐induced EMT process in endometrial cells.

Epithelial‐mesenchymal transition in endometrial cells is important for endometriosis establishment.^11^ As a reproductive tissue, endometrial cells located in high level of oestrogen. Thus, endometriosis has been considered as an oestrogen‐dependent disease.^18^ Many studies demonstrated that oestrogen could induce in many cancers and adenomyosis.^19^, ^20^, ^21^ Recent studies also suggested that oestrogen promotes EMT process during the development of endometriosis.^7^, ^10^ However, the relationship between dysregulated circRNAs and oestrogen‐induced EMT in endometriosis remain largely unknown. Here, our results showed that the expression of circ_0004712 was significantly increased in endometrial epithelial cells after E_2_ treatment. Meanwhile, knock‐down the expression of circ_0004712 could markedly suppress cell migration activity in endometrial cells. These results revealed that circ_0004712 involved in regulating the E_2_‐induced EMT process.

circRNAs containing the target site could bind to specific miRNAs to inhibit the expression and function of miRNAs.^22^ In this work, circ_0004712 act as a sponge of miR‐148a‐3p. Up‐regulating the expression of miR‐148a‐3p could suppress cell migration as well as circ_0004712 knocking down. Recent studies suggested that overexpression of miR‐148a‐3p could suppress the process of EMT in cancer cells.^23^, ^24^, ^25^ Thus, circ_0004712 might sponge miR‐148a‐3p to promote EMT during E_2_ treatment in endometrial cells.

miRNAs regulate the cellular process by suppressing the expression of target genes via directly binding to the 3’‐UTR of the genes.^26^ In our study, we demonstrated SOS2 was a target gene of miR‐148a‐3p involved in the E_2_‐induced EMT process. SOS2 is a number of SOS (Son of sevenless) family, which is the Ras‐specific guanine nucleotide‐exchange factor. SOS2 enhances the activation of Ras and regulates many cellular process, such as cell proliferation and migration.^27^ Previous study suggested that inhibiting the expression of SOS2 could suppress the cell proliferation and EMT process by down‐regulating MAPK/Erk pathway in non‐small‐cell lung cancer cells.^15^ In our study, we also found the expression of SOS2 increased in endometrial cells after E_2_ treatment and demonstrated the binding ship between SOS2 and miR‐148a‐3p. Furthermore, inhibiting the expression of SOS2 could suppress cell migration after E_2_ treatment. Thus, SOS2 acted as the target gene of miR‐148a‐3p involved in the E_2_‐induced EMT process in endometrial cells.

The up‐regulation of N‐cadherin and Vimentin and down‐regulation of E‐cadherin are the most influential biomarkers of EMT.^8^ E_2_ have been demonstrated to reduce the expression of E‐cadherin ^28^ and activate β‐catenin signalling to promote EMT process.^29^ Our results also revealed E_2_ could activate β‐catenin signalling. Furthermore, knocking down the expression of circ_0004712 and over‐expression of miR‐148a‐3p suppressed activation of β‐catenin signalling, subsequently up‐regulated E‐cadherin expression and down‐regulate N‐cadherin expression after E_2_ treatment in endometrial epithelial cells. Reducing the expression of miR‐148a‐3p could recover the effect of circ_0004712 knocking down. Furthermore, we also found inhibiting the expression of SOS2 could recover the effect of miR‐148a‐3p knocking down on the expression of N‐cadherin and E‐cadherin. However, the precise relationship between SOS2 and β‐catenin signalling needs more experiments to illustrate.

In conclusion, this work demonstrated E_2_ could promote EMT to enhance cell migration in the development of endometriosis by up‐regulating circ_0004712 expression levels. Circ_0004712 sponged miR‐148a‐3p to activate the β‐catenin signalling to promote EMT process. These findings revealed the important effect of circRNAs on endometriosis and provided new biomarker for early diagnosis and therapeutic targets for the treatment of endometriosis.

## CONFLICTS OF INTEREST

The authors declare no conflict of interest.

## AUTHOR CONTRIBUTION

**Xin He:** Methodology (equal); Visualization (equal); Writing‐original draft (lead). **Nana Liu:** Investigation (equal); Methodology (equal); Visualization (equal); Writing‐original draft (supporting). **Tianyi Mu:** Formal analysis (equal); Investigation (equal); Software (equal). **Dan Lu:** Data curation (equal); Investigation (lead). **Chanwei Jia:** Formal analysis (equal); Investigation (equal). **Yushu Wang:** Formal analysis (equal); Software (equal). **Chenghong Yin:** Formal analysis (equal); Software (equal). **Lingyan Liu:** Validation (equal); Visualization (equal). **liying zhou:** Validation (equal); Visualization (equal). **Xiaowu Huang:** Conceptualization (supporting); Funding acquisition (equal); Resources (equal); Supervision (lead); Validation (lead); Writing‐review & editing (supporting). **Yanmin Ma:** Conceptualization (supporting); Funding acquisition (equal); Project administration (lead); Resources (equal); Supervision (lead); Writing‐review & editing (lead). 

## Data Availability

The data that support the findings of this study are available from the corresponding author upon reasonable request.
